# Primary percutaneous coronary intervention during ST elevation myocardial infarction in prosthetic valve endocarditis: a case report

**DOI:** 10.1186/s12872-018-0750-3

**Published:** 2018-02-09

**Authors:** Alfonso Campanile, Guido Tavazzi, Francesco Caprioglio, Fausto Rigo

**Affiliations:** 1 Department of Cardiology, Hospital “S. M. della Misericordia”, Perugia, Italy; 2 Department of Anaesthesia, Intensive Care and Pain Therapy, Fondazione Policlinico San Matteo IRCCS, University of Pavia, Pavia, Italy; 3 Department of Cardiology, Dell’Angelo Hospital, Mestre, Italy

**Keywords:** AMI, pPCI, Endocarditis, TTE/TEE, Elderly patients

## Abstract

**Background:**

Infective endocarditis (IE) is still a condition with high mortality and morbidity, especially in the elderly, and in patients with prosthetic valves. The concept of “time-to-therapy” plays a key role for the prompt management of IE and related complications, and the currently available multimodality imaging may play a key role in this setting. Myocardial ischemia due to extrinsic coronary compression from an aortic abscess is an extremely rare condition where the optimal therapeutic strategy has not been defined yet. We present herein the first case of a patient with ST elevation myocardial infarction caused by an aortic root abscess treated with percutaneous stent implantation.

**Case presentation:**

An 82-year-old woman with a history of atrial fibrillation, chronic renal failure, anemia and a bioprosthetic aortic valve replacement performed in 2014, was admitted to hospital with profound asthenia and a pyrexia of unknown origin. Because of high clinical suspicion of endocarditis, a trans-esophageal echocardiogram was performed. Empirical broad-spectrum antimicrobial therapy was initiated, followed by targeted treatment based on the results of blood cultures (*Staphylococcus aureus*). The echocardiogram did not show vegetations and the patient was managed conservatively. She suddenly deteriorated, due to an acute coronary syndrome (ACS) with anterior ST segment elevation. An urgent angiogram was performed, and extrinsic compression of the left coronary system, due to an aortic root abscess, was suspected. After discussion with the surgical team, percutaneous revascularization was attempted, aiming to restore satisfactory hemodynamics, in order to plan surgery. Unfortunately, the patient rapidly developed cardiogenic shock, with multi organ failure, and died in less than 24 h.

**Conclusions:**

Patients with fever, and significant risk factors for endocarditis, who develop ACS, need a prompt diagnostic work up, including trans-esophageal echocardiography. At present, the specific timing of echocardiographic follow-up and surgical intervention is still a matter of debate, and our case aims to highlight the importance of this aspect in the management of endocarditis, in order to avoid severe complications that adversely affect patient prognosis.

**Electronic supplementary material:**

The online version of this article (10.1186/s12872-018-0750-3) contains supplementary material, which is available to authorized users.

## Background

Acute coronary syndrome (ACS) occurs in only 1–3% of patients with infective endocarditis and the most appropriate management is therefore unknown [1, 2].

The possible mechanisms responsible for myocardial ischemia in endocarditis are the presence of preexisting coronary artery disease, coronary emboli from aortic vegetations, obstruction of the coronary ostium due to a large vegetation, severe aortic insufficiency and external coronary artery compression [3–5]. The latter is a rare and lethal finding with few reported cases available in the medical literature [3, 5–14].

We report a case of primary percutaneous coronary intervention (pPCI) performed during ST segment elevation myocardial infarction (STEMI) in the setting of a bioprosthetic aortic root abscess compressing the left main coronary artery. In previous similar reports, a surgical intervention represented the main therapeutic approach [5, 8, 13]. We believe that sharing experiences can be of help in defining a common procedural strategy in such rare conditions.

## Case presentation

**Fig. 1:**
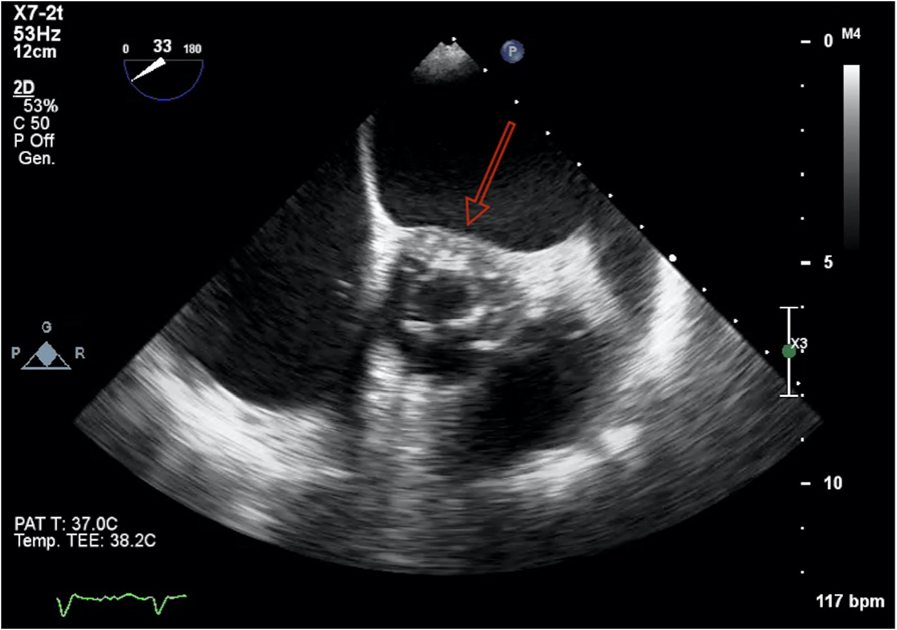
Trans-esophageal short axis view of the aorta showing the aortic bioprosthesis in a correct position, and a pronounced aortic root thickening (marked with the red arrow in the picture)

**Fig. 2:**
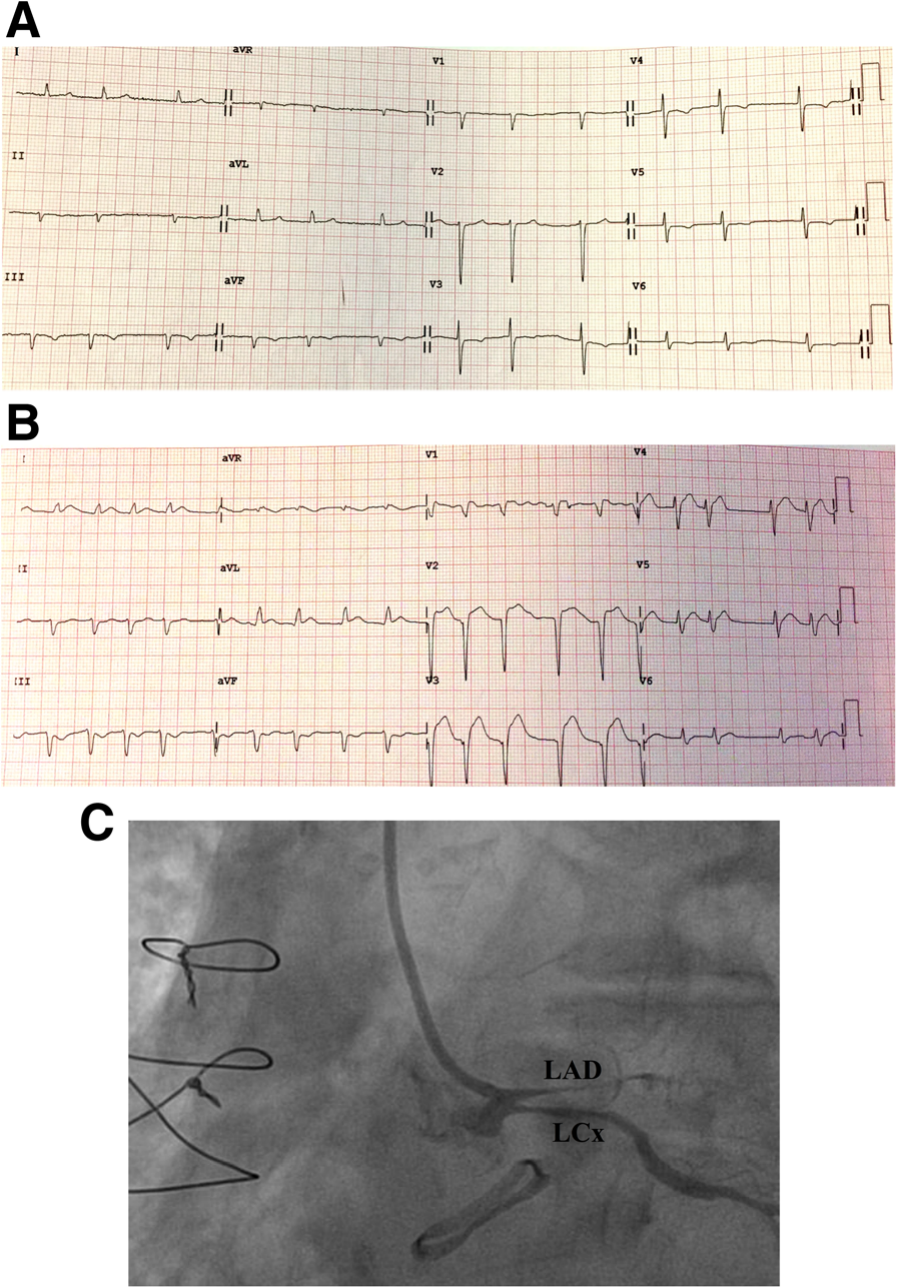
**a**, **b** and **c** (from the top to the bottom): **a** basal ECG showing atrial fibrillation with T-wave inversion in the inferior and anterolateral leads. **b** ECG acquired during chest pain showing anterior ST elevation. **c** The left coronary angiogram, showing occlusion of the proximal LAD and a long, tubular stenosis of the proximal LCx

**Fig. 3:**
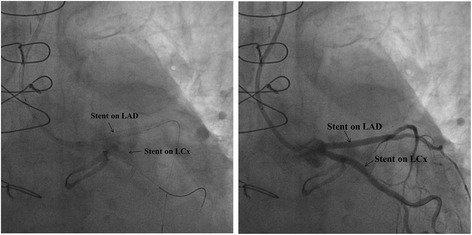
The stent implantation procedure in the left coronary system before (on the left) and after (on the right) contrast injection; stents and coronary vessels involved are marked with black arrows

An 82-year-old woman with a history of atrial fibrillation, chronic renal failure, anemia and a bioprosthetic aortic valve replacement performed in 2014, was admitted to hospital with profound asthenia and a pyrexia of unknown origin (PUO). No coronary lesions were detected at the time of aortic valve replacement. Clinical parameters and laboratory findings on admission are summarized in Table 1. The chest X-ray did not show signs of consolidation. Samples of urine, blood and sputum were sent for microbiological screening. A trans-esophageal echocardiogram (TEE) was performed as part of the PUO diagnostic work-up [15] and showed the left ventricle was not dilated with normal global systolic function, moderate mitral and tricuspid regurgitation, normal bioprosthesic function but pronounced aortic root thickening (Fig. 1 and Additional file 1: Video S1). Because of high clinical suspicion of endocarditis, empirical broad spectrum antimicrobial therapy was initiated, followed by targeted treatment (clyndamicin 600 mg three times daily) based upon the results of blood cultures (*Staphylococcus aureus*). The initial patient response was satisfactory. She became afebrile and the inflammatory markers improved. A conservative strategy was planned. During her first week in hospital she underwent a routine transthoracic echocardiogram (TTE) and a second TEE was also repeated, 7 days later, with no new findings. Due to the stable clinical conditions, the initial management plan remained unchanged. It was aimed to complete 1 month of antibiotic therapy before performing a new TEE. However, almost 1 month after her admission, a new rise in her inflammatory markers and temperature was observed associated with clinical deterioration. 24 h later the patient developed central chest discomfort and an ECG showed new ST segment elevation in the anterior precordial leads (Fig. 2a and b). Immediate coronary angiography was performed. A severe narrowing, followed by complete occlusion of the left anterior descending coronary artery (LAD) and a critical, long tubular stenosis of the proximal left circumflex artery (LCx), was detected (Fig. 2c, Additional file 2: Video S2). The interventional cardiologist suspected that there was extrinsic compression of the coronary arteries from the aortic root abscess (Additional file 3: Video S3). The surgical team was involved, however due to the unstable hemodynamic condition (blood pressure: 80/50 mmHg, heart rate-HR: 110 bpm, pO2: 8 kPa in high flow oxygen treatment), it was decided to attempt percutaneous treatment. Two bare metal stents (BMS-Rebel Boston Scientific®) were implanted respectively in the proximal segment of the LAD (3.0 * 24 mm) and the LCx (3.5 * 20 mm) (Fig. 3, Additional files 2, 3, 4, 5, 6, 7, 8 and 9: Videos S2-S9).

**Table 1:** ᅟ

*Clinical parameters*	
Temperature (°C)	38,2
Blood pressure (mmHg)	100/50
Mean heart rate (bpm)	88
Peripheral oxygen saturation in room air (%)	95
*Laboratory findings*	
Hemoglobin (g/L)	108
Hematocrit (%)	30,5
Mean corpuscular volume (MCV, fl)	89
Platelet count (× 10^9^/L)	162
White cell count (× 10^9^/L)	18,7
International normalized ratio (INR)	1,41
Creatinine (μmol/L)	169,8
Azotemia (mmol/L)	43,6
Potassium (mmol/L)	3,5
Total bilirubin (μmol/L)	23,9
C-reactive protein (nmol/L)	916,2
Procalcitonin (μg/L)	0,93

**Fig. 4:**
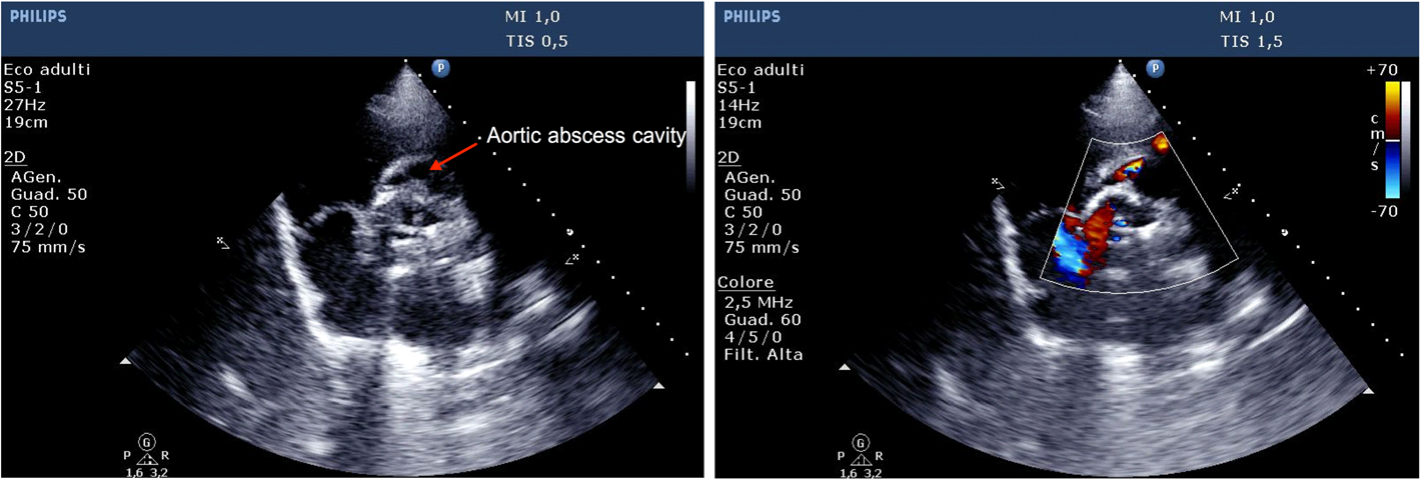
Trans-thoracic short axis view of the aorta in bi-dimensional (on the left) and color mode representation (on the right), showing a cavity around the aortic root, highly suspicious for a peri-prosthetic abscess

On admission to the coronary care unit the patient was in cardiogenic shock requiring inotropes, vasopressors and non-invasive ventilation. The biochemical results showed a white blood cell count (WBC) of 25,7 × 10^9^/L, creatine kinase-MB fraction 184 μg/L, and Troponin I (TnI) > 70.000 μg/L. A transthoracic echocardiogram was performed (Fig. 4, Additional files 10, 11, 12, 13 and 14: Videos S10-S14) with evidence of severe biventricular dysfunction, severe mitral and tricuspid regurgitation, and significant tissue damage in the aortic prosthetic region, where a cavity was also identified, suggesting an abscess. Despite intensive treatment the patient died within 24 h.

## Discussion

We describe a rare complication of prosthetic valve endocarditis: an aortic root abscess causing external coronary artery compression and acute myocardial infarction. Although a few reports of myocardial infarction during infective endocarditis involving a prosthetic valve exist in the literature, our case shows an unusual complication and treatment strategy.

Prosthetic valve endocarditis is associated with a high in-hospital mortality (more than 40%) [16, 17] and when complicated by an ACS, the morbidity and mortality rates further increase [18]. In the management algorithm presented by Manzano et al., when a patient presents with ACS and suspected endocarditis, a TEE should be performed and angiography, with percutaneous reperfusion, should only be considered if a ST segment elevation is present on the ECG in the absence of periannular complication [1]. In our case, an immediate coronary angiogram was performed because ST elevation myocardial infarction, complicated by haemodynamic instability, was evident, while the abscess cavity was only suspected. The hypothesis of external coronary compression due to periannular complication was, indeed, only formulated at the time of the invasive procedure due to the specific angiographic pattern (diffuse narrowing of the coronary arteries with severe stenosis of the proximal segments) along with the peculiar clinical presentation [19]. However, an erosion/perforation of the abscess into left coronary artery, could not be definitely ruled out. Some images, indeed, showed enrichment of the contrast medium in the abscess during angiography (Additional files 2 and 3: Video S2 and S3).

After a discussion with the surgical team, a two-step approach, with percutaneous revascularization first, aiming for restoration of stable hemodynamics, followed by a surgical procedure, was attempted, similar to the case described by Sugi K et al. [20].

The outcome in this setting is strongly affected by several different variables (grade of inflammatory response, pathogen causing endocarditis, sepsis dissemination, site of coronary obstruction and the grade of valve dysfunction) [20] and, unfortunately, our patient rapidly developed multi organ failure.

## Conclusions

Considering the paucity of data available, the management of each patient presenting with ST elevation and valve/prosthetic endocarditis should be individualized and a multidisciplinary discussion should be advocated.

Patients with fever, and significant risk factors for endocarditis, who develop ACS, need a prompt diagnostic work up, including trans-esophageal echocardiography. The specific timing of echocardiographic follow-up and surgical intervention is still a matter of debate [17, 21], and our case aims to highlight the importance of this aspect in the management of endocarditis, in order to avoid severe complications that adversely affect patient prognosis.

## Additional files

Additional file 1: Video S1. (Trans-esophageal short axis view of the aorta): the clip shows a normal opening of the aortic bioprosthesis and a pronounced aortic root thickening. (MOV 702 kb)

Additional file 2: Video S2. Clip of the interventional procedure performed to establish a satisfactory flow on the left coronary system. (Left coronary angiography): the diagnostic left coronary angiogram showing a severe narrowing, followed by complete occlusion of the left anterior descending coronary artery (LAD) and a critical, long tubular stenosis of the proximal left circumflex artery (LCx). (MOV 1760 kb)

Additional file 3: Video S3. Clip of the interventional procedure performed to establish a satisfactory flow on the left coronary system. (Ascending aortic angiography): the ascending aortic angiogram showing a possible, extrinsic compression, of the coronary arteries from an aortic root abscess. The clip also shows enrichment of the contrast medium in the abscess during angiography so that, an erosion/perforation of the abscess into left coronary artery, can’t be definitely ruled out. (MOV 3090 kb)

Additional file 4: Video S4. Clip of the interventional procedure performed to establish a satisfactory flow on the left coronary system. (Wires advancement in the left coronary system): the clip shows the wires that have been introduced in the LAD and LCx. (MOV 3720 kb)

Additional file 5: Video S5. Clip of the interventional procedure performed to establish a satisfactory flow on the left coronary system. (After only balloon angioplasty): video showing the result soon after POBA. (MOV 3030 kb)

Additional file 6: Video S6. Clip of the interventional procedure performed to establish a satisfactory flow on the left coronary system. (Stent deployment in the LAD). (MOV 1130 kb)

Additional file 7: Video S7. Clip of the interventional procedure performed to establish a satisfactory flow on the left coronary system. (Post stent implantation in the LAD): videos showing the stent deployment in the LAD and the result of the procedure. (MOV 1890 kb)

Additional file 8: Video S8. Clip of the interventional procedure performed to establish a satisfactory flow on the left coronary system. (Stent deployment in the LCx artery). (MOV 511 kb)

Additional file 9: Video S9. Clip of the interventional procedure performed to establish a satisfactory flow on the left coronary system. (Final result): videos showing the stent deployment in the LCx and the final procedural result. (MOV 2800 kb)

Additional file 10: Video S10. Clip of the trans-thoracic examination. (Parasternal long axis view): evidence of severe left ventricle dysfunction. (M4V 371 kb)

Additional file 11: Video S11. Clip of the trans-thoracic examination. (Parasternal long axis view with color): the clip shows a significant tissue damage in the aortic prosthetic region, where a cavity is also identified, suggesting an abscess. (M4V 328 kb)

Additional file 12: Video S12. Clip of the trans-thoracic examination. (Parasternal short axis of the aortic valve without and with color mode representation): different view of the significant tissue damage in the aortic prosthetic region. (MP4 5930 kb)

Additional file 13: Video S13. Clip of the trans-thoracic examination. (Apical four chamber view without and with color mode representation): evidence, respectively, of severe biventricular dysfunction, severe TR and MR. (MP4 8680 kb)

Additional file 14: Video S14. Clip of the trans-thoracic examination. (Subcostal view): view showing a dilated inferior vena cava and right pleural effusion. (M4V 367 kb)

## Abbreviations

ACS: Acute coronary syndrome
BMS: Bare metal stent
IE: Infective endocarditis
ECG: electrocardiogram
LAD: left anterior descending coronary artery
LCx: left circumflex artery
POBA: plain old balloon angioplasty
TR: tricuspid regurgitation
MR: mitral regurgitation
pPCI: Primary percutaneous coronary intervention
PUO: Pyrexia of unknown origin
STEMI: ST segment elevation myocardial infarction
TEE: Trans-esophageal echocardiogram
TTE: Transthoracic echocardiogram
WBC: White blood cell count
